# Systematic review of the role of angiopoietin-1 and angiopoietin-2 in *Plasmodium* species infections: biomarkers or therapeutic targets?

**DOI:** 10.1186/s12936-016-1624-8

**Published:** 2016-12-01

**Authors:** Gerdie M. de Jong, Jasper J. Slager, Annelies Verbon, Jaap J. van Hellemond, Perry J. J. van Genderen

**Affiliations:** 1 Institute for Tropical Diseases, Harbour Hospital, Haringvliet 2, Rotterdam, The Netherlands; 2 Department of Medical Microbiology and Infectious Diseases, Erasmus MC, Rotterdam, The Netherlands

**Keywords:** Malaria, Angiopoietin-1, Angiopoietin-2, Endothelial cell activation, Biomarker, Therapeutic target

## Abstract

**Background:**

Levels of both angiopoietin-1 (Ang-1) and angiopoietin-2 (Ang-2) correlate with malaria disease severity and are proposed as biomarkers and possible therapeutic targets. To establish their role in malaria, a systematic review was performed of the literature on Ang-1 and Ang-2 with regard to their potential as biomarkers in malaria and discuss their possible place in adjuvant treatment regimens.

**Methods:**

Ten electronic databases were systematically searched to identify studies investigating Ang-1 and Ang-2 in human and murine malaria in both clinical and experimental settings. Information about the predictive value of Ang-1 and Ang-2 for disease severity and their regulatory changes in interventional studies were extracted.

**Results:**

Some 579 studies were screened; 26 were included for analysis. In all five studies that determined Ang-1 levels and in all 11 studies that determined Ang-2 in different disease severity states in falciparum malaria, a decline in Ang-1 and an increase of Ang-2 levels was associated with increasing disease severity. All nine studies that determined angiopoietin levels in *Plasmodium falciparum* patients to study their ability as biomarkers could distinguish between multiple disease severity states; the more the disease severity states differed, the better they could be distinguished. Five studies differentiating malaria survivors from non-survivors with Ang-2 as marker found an AUROC in a range of 0.71–0.83, which performed as well or better than lactate. Prophylactic administration of FTY720, rosiglitazone or inhalation of nitric oxide (NO) during malaria disease in mice resulted in an increase in Ang-1, a decrease in Ang-2 and an increased survival. For rosiglitazone, a decrease in Ang-2/Ang-1 ratio was observed after post-infection treatment in mice and humans with malaria, but for inhalation of NO, an effect on Ang-1 and survival was only observed in mice.

**Conclusion:**

Both Ang-1 and Ang-2 levels correlate with and can distinguish between malaria disease severity states within the group of malaria-infected patients. However, distinct comparisons of disease severity states were made in distinct studies and not all distinctions made had clinical relevance. Changes in levels of Ang-1 and Ang-2 might also reflect treatment effectiveness and are promising therapeutic targets as part of multi-targeted therapy.

**Electronic supplementary material:**

The online version of this article (doi:10.1186/s12936-016-1624-8) contains supplementary material, which is available to authorized users.

## Background

### General aspects of malaria and endothelial cell activation

Even though many disease control interventions have been implemented, malaria remains a major health problem with an estimated 214 million cases and 438,000 deaths worldwide in 2015 [1]. Most of these malaria deaths were caused by *Plasmodium falciparum*, one of the six *Plasmodium* species (*Plasmodium cynomolgi*, *P. falciparum*, *Plasmodium knowlesi*, *Plasmodium malariae*, *Plasmodium ovale*, *Plasmodium vivax*) that can infect humans. The high pathogenicity of *P. falciparum* is in part related to the expression of *P. falciparum* erythrocyte membrane protein-1 (*Pf*EMP-1) on the membranes of infected erythrocytes. The infected erythrocytes with *Pf*EMP-1 on the membrane adhere to the vascular endothelium of vital organs. This process is called sequestration and will cause a partial obstruction of the blood flow. Together with the increased deformability of uninfected erythrocytes and adherence of uninfected erythrocytes to infected erythrocytes, which is called rosetting [2], extensive sequestration will lead to a decline in oxygen delivery to the organs. Without treatment, the lack of oxygen will result in acidosis and multi-organ failure, along with endothelial barrier dysfunction and inflammation. This may eventually lead to death in untreated cases, especially in non-immune individuals. Of the non-falciparum species, *P. vivax,* which may run a relatively benign course compared to *P. falciparum*, is most important and can also cause severe malaria and result in death. The pathogenesis of *P. vivax* infection differs from *P. falciparum* infection and is still poorly understood. *Plasmodium vivax* infects only young erythrocytes (reticulocytes) which can also adhere to the vascular endothelium, but showed ten times less cyto-adhesion compared to *P. falciparum*-infected erythrocytes in vitro [3]. Also, the cytokine response differs between the species: *P. vivax* showed a stronger response than *P. falciparum* [4]. Whether cyto-adherence in *P. vivax* leads to disproportional organ-specific parasitaemia and what role the cytokine response plays in the pathogenesis remains unclear.

### Endothelial cell activation

Endothelial cell activation is crucial in the pathogenesis of *P. falciparum* infection as *Pf*EMP-1 on the erythrocyte membranes interacts with several cyto-adherence receptors that show a low, basal expression pattern in a non-inflammatory environment, but are upregulated during malaria infection [5]. Activation of endothelial cells is complex and interconnected with other processes, such as coagulation and inflammation. Quiescent endothelial cells have anticoagulant properties due to expression of several proteins, such as protein C and thrombomodulin. On the other hand, after activation by, e.g., thrombin [6], fibrin [7, 8], complement factors C5–C9 [9], or platelets [10], endothelial cells express or release procoagulant and inflammatory proteins, such as tissue factor, von Willebrand factor (vWF), Ang-2, I-CAM, V-CAM, and E-selectin [5, 11]. Endothelial cell activation occurs not only during malaria but also during many other infectious diseases, such as bacterial sepsis [12] and dengue haemorrhagic fever [13, 14]. To study endothelial cell activation during infectious diseases, expression levels of multiple endothelial-cell activation markers have been determined and were found to be increased, including the vascular growth factors Ang-1 and Ang-2, which were extensively studied during systemic inflammation (reviewed in [15–20]). Their role in endothelial cell activation during malaria has also been investigated.

### Ang-1, Ang-2 and endothelial cell activation

Ang-1 and Ang-2 are ligands of the Tie-2 receptor, which is expressed on endothelial cells and regulates endothelial quiescence during normal physiological conditions. Ang-1 is constitutively produced and excreted into the blood by pericytes and smooth muscle cells and it is also stored in platelets. Ang-1 binds to the Tie-2 receptor, thereby acting as an agonist. Upon Ang-1/Tie-2 interaction, the most important downstream signalling event is protein kinase B phosphorylation by phosphoinositide-3-kinase resulting in an anti-apoptotic and anti-inflammatory status of the endothelial cell.

**Fig. 1:**
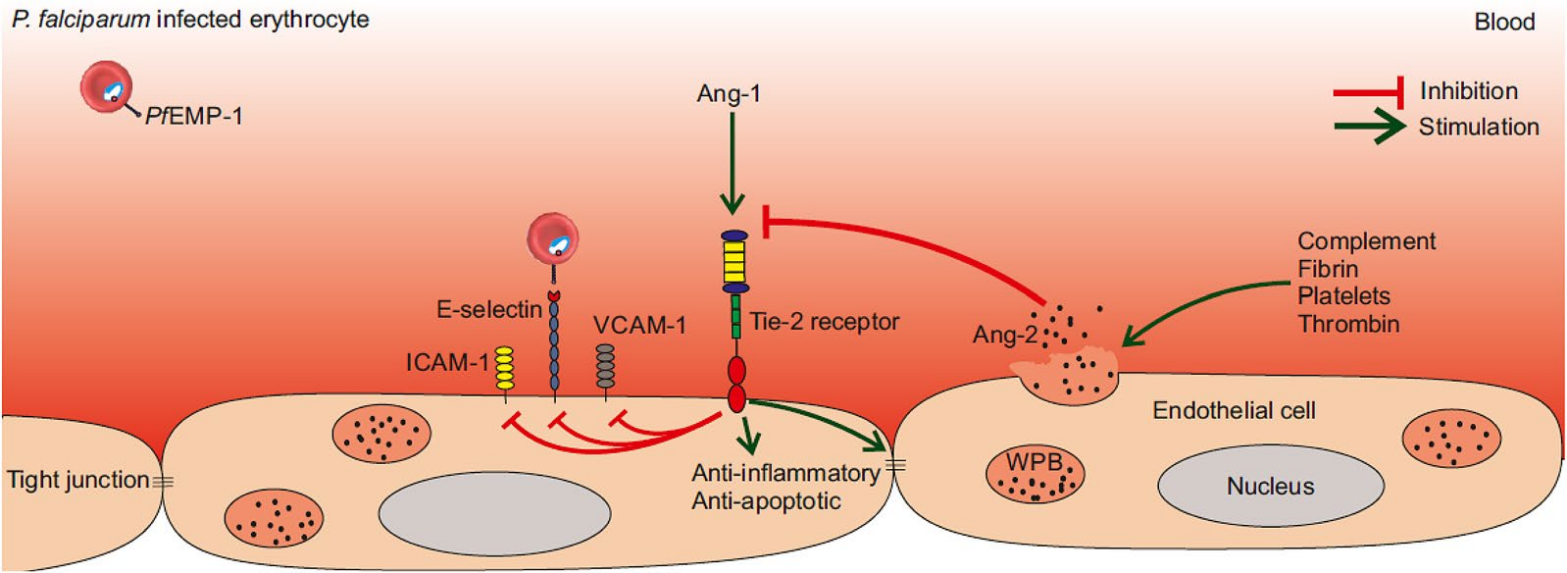
Schematic overview of function and localisation of Ang-1, Ang-2 and the Tie-2 receptor. Ang-2 is pre-stored in the Weibel–Palade bodies in endothelial cells and is released upon endothelial cell activation. Ang-2 replaces Ang-1 by binding the Tie-2 receptor, preventing its activation and thereby blocking the anti-inflammatory, anti-apoptotic and tight-junction supporting effects of Ang-1. Ang-1, angiopoietin-1; Ang-2, angiopoietin-2; ICAM-1, E-selectin, VCAM-1, adhesion molecules; *Pf*EMP-1, *P. falciparum* erythrocyte membrane protein 1; WPB, Weibel–Palade body

Ang-2 is produced in endothelial cells and pre-stored in the Weibel–Palade bodies (WPB) together with vWF. Upon activation of endothelial cells, exocytosis of the WPB is induced and their content is released into the blood stream [21]. The increased Ang-2/Ang-1 ratio causes a replacement of the Ang-1-Tie-2 interaction by an Ang-2-Tie-2 interaction. In the presence of Ang-1, Ang-2 acts as a functional antagonist and the Ang-2-Tie-2 interaction results in the blocking of the protective, anti-inflammatory and anti-apoptotic effect of Ang-1 (Fig. 1). Therefore, Ang-2 release and binding to Tie-2 receptors further stimulates the inflammatory response of endothelial cells. However, an agonistic function of Ang-2 has also been described and is influenced by cell type and experimental conditions [22]. A decrease in the Ang-1 concentration during pro-inflammatory conditions, also contributes to the increased Ang-2/Ang-1 ratio. The underlying mechanisms of the Ang-1 decrease have not been defined yet. From these findings, it may be hypothesized that the role of Ang-1 is protective during malaria and the role of Ang-2 may be harmful.

### Ang-1 and Ang-2 are likely to play a role early in malaria pathogenesis

During malaria, a decrease in Ang-1 and an increase in Ang-2 concentration have been observed [15]. It is not known whether the decrease of Ang-1 and increase of Ang-2 are the result of the endothelium activation status or that the altered levels also contribute to endothelium activation. However, during the initial processes that lead to pathology in malaria, Ang-1 and Ang-2 might play a role as (1) Ang-1 and Ang-2 levels strongly correlate with disease severity and mortality [15]; (2) in sepsis, which shares many similarities with severe malaria [15, 18, 23], interfering in the angiopoietin-Tie-2 system, had a huge effect on the inflammatory response, vascular leakage and survival [24]; and, (3) Ang-2 can be released rapidly upon activation of endothelial cells as it is pre-stored in WPBs [21]. After exocytosis of WPBs, released Ang-2 may then have extended effects in further activation of endothelial cells by activation of a pro-inflammatory amplification loop.

Because of the correlation of Ang-1 and Ang-2 levels with disease severity and mortality, Ang-1 and Ang-2 have been evaluated as biomarkers for malaria disease severity in multiple studies. However, each individual study compared different disease severity states and the studies were performed in distinct populations at different geographical locations around the world. To gain more insight in their role during malaria, the current literature concerning the use of Ang-1 and Ang-2 as biomarker in a clinical setting is here reviewed systematically. Furthermore, the role of Ang-1 and Ang-2 as potential therapeutic targets in malaria is evaluated as they might play a causative role in the pathogenesis.

## Methods

### Literature search and inclusion criteria

A broad systematic literature-search strategy was created combining synonyms for angiopoietin with synonyms for malaria or *Plasmodium*. In Embase, Medline and CInahl, thesaurus terms were used together with words in title and/or abstract. In the other databases only titles and/or abstracts were searched. The databases Embase.com, Medline (Ovid), Cochrane Central registry of trials, Web of Science, Scopus, cinahl (EBSCOhost), Pubmed as supplied by publisher (for the most recent, not yet indexed, articles), lilacs, scielo, ProQuest, and Google scholar (an additional search), were searched without data limit, from inception lastly on 15 June 2016 (see Additional file 1). Based on the titles and abstracts, potentially relevant articles were selected by two independent reviewers (GMdJ, JJS) for full text screening. Articles in English meeting the inclusion criteria were included (see Additional file 2). In case of disagreement between the reviewers, a third independent reviewer was consulted (JJvH).

Reported studies were included if Ang-1 and/or Ang-2 protein or mRNA levels were determined in any specimen in humans and/or mice with a confirmed *Plasmodium* infection. Through tracking the citations in selected articles, no additional articles were found.

### Data extraction

The selected articles were grouped, depending on the study aim, into: (I) studies performed in non-pregnant humans; and, (II) interventional studies. The aim of studies in I was to correlate Ang-1 and Ang-2 levels with disease severity and in II to find an adjuvant therapy next to anti-malarial treatment.

For the articles of I, information was extracted on the *Plasmodium* species, the study population, the Ang-1 and/or Ang-2 protein and/or mRNA levels or the Ang-2/Ang-1 ratio in various disease states, and information about the associations of Ang-1 and Ang-2 levels with other variables. In the studies included in II, data were extracted regarding study subject, mice species or population characteristics, details of the studied intervention, Ang-1, Ang-2 protein or mRNA levels and the Ang-2/Ang-1 ratio.

## Results

### Characteristics of included studies

Twenty-six studies assessing Ang-1 and/or Ang-2 protein and/or mRNA levels in human and/or mice with a confirmed *Plasmodium* infection were identified and included for analysis; 22 studies were performed in humans only, of which five included children and adults in Asia and Canada as a single group (see Additional file 3), six studies included adults only and were performed in Asia and Brazil (see Additional file 4), eight studies included children only and were all performed in only African countries (see Additional file 5) and one study included a separate group of children and adult [25] (see Additional files 4, 5). Three studies provided data on mice only (see Additional file 6). One interventional study combined results of studies in mice and humans [26] (see Additional file 6).

In nine studies a discriminative value of Ang-1, Ang-2 levels or the Ang-2/Ang-1 ratio for disease severity was reported [25, 27–35] (see Additional file 7). Four studies determined the Ang-1 and/or the Ang-2 level in humans after start of anti-malarial treatment [29, 34, 36, 37]. Three studies investigated the effect of adjuvant therapy next to standard treatment on survival and on Ang-1 and/or Ang-2 levels in mice [38–40], two in humans [41, 42] and one in both humans and mice [26] during *Plasmodium* species infection (see Additional file 6). One study used a mouse model with one deleted Ang-1 allele [26] (see Additional file 6).

### Aim I: Ang-1 and Ang-2 levels as biomarkers for malaria disease severity

#### Ang-1 is decreased and Ang-2 is increased during *Plasmodium* species infection in non-pregnant humans

In ten out of eleven studies comparing healthy controls with either uncomplicated [25, 32, 34, 35, 37, 43–45], severe [34, 35, 43, 45, 46] or cerebral falciparum malaria [25, 32, 37, 44, 45] or uncomplicated [43, 47] or severe [43] vivax malaria, a similar pattern in changes was observed for Ang-1 and Ang-2 levels: a decrease of Ang-1 [25, 32], an increase of Ang-2 [25, 32, 34, 35, 37, 43–47] and consequently an increase of the Ang-2/Ang-1 ratio [25, 32] (see Additional files 3, 4, 5). Adults and children, living either in endemic areas or travellers in non-endemic areas all showed these similar changes. However, one study [36] out of three [25, 32, 36] that determined the Ang-1 levels in falciparum malaria found an increase in Ang-1 compared with the levels in healthy controls opposed to a decline in the other studies. The absolute concentration of Ang-2 was significantly higher in *P. vivax*-infected patients compared to *P. falciparum*-infected patients in Indonesia, independent of the observed peripheral parasitaemia [47]. Contrastingly, a significant lower Ang-2 concentration was observed in *P. vivax*- compared to *P. falciparum*-infected travellers in Canada [48]. Barber et al. [43] determined also the Ang-2 levels in both uncomplicated vivax malaria [median pg/ml (IQR)] [4557 (3463–6197)] and falciparum malaria [3230 (2123–5243)] and in severe vivax malaria [8857 (6547–9734)] and falciparum malaria [8371 (3963–13,463)]. However, this study did not statistically compare the levels between the vivax and falciparum malaria, although Ang-2 seems to be higher in *P. vivax*-infected patients especially when the higher parasitaemia of *P. falciparum*-infected patients was taken into account.

Differences in the absolute Ang-1 and Ang-2 concentrations were also found between disease severity states: lower Ang-1 and higher Ang-2 concentrations were observed in severe disease compared to uncomplicated disease in both falciparum [25, 28–30, 32, 34, 43–45] and vivax malaria [31, 43] and in non-survivors compared to survivors infected with *P. falciparum* [25, 27, 30, 32–35, 41, 46] (see Additional files 3, 4, 5, 6).

Furthermore, an elevated Ang-2 concentration was associated with respiratory distress [49], impaired consciousness [49], acute kidney injury [33, 46], multi-organ failure [46], anaemia [33], jaundice [33], hypoglycaemia [33], pure cerebral malaria (cerebral malaria patients without the other complications of severe malaria according to WHO 2000 criteria) [33] and higher Ang-1 levels were associated with a higher platelet count in *P. falciparum* and *P. vivax* infections [31, 33, 50].

#### Ang-1 increases and Ang-2 decreases after start of anti-malarial treatment in survivors

An increase in Ang-1 and a decrease in Ang-2 levels in survivors compared to non-survivors was observed after the start of treatment in both children [29] and adults [34, 36] (see Additional files 3, 4, 5) and one study found an average Ang-2 decrease of 2.7 ng/ml over a 24-hour period in survivors [34]. Moxon et al. [37] found a nearly two-fold decline in Ang-2 level in children with uncomplicated malaria and a five-fold decline in children with severe malaria 28 days after the start of treatment. However, 28 days after treatment, the Ang-2 levels in uncomplicated malaria were still significant higher than Ang-2 levels in healthy controls. This lack of decrease to baseline levels might be caused by a 50% loss to follow-up, which may have led to a biased difference in Ang-2 value between healthy control and patients after treatment.

#### Ang-1 and Ang-2 levels have a varying discriminative power for distinguishing disease severity states in humans

Uncomplicated falciparum malaria can rapidly progress into life-threatening disease due to the exponential increase in parasite biomass and sequestration of infected erythrocytes, which may lead to organ failure. The currently used biomarkers for disease severity have a limited predictive value to identify those patients that will rapidly progress towards complicated disease [51]. Several new markers have been proposed and investigated [52] as prognostic parameters for severe malaria, including Ang-1 and Ang-2. In distinct studies Ang-1, Ang-2 and the Ang-2/Ang-1 ratio were used to investigate whether they could distinguish different disease severity states and survivors from non-survivors [25, 27–35] (see Additional file 7).

The Ang-1 concentration was found to be the best discriminator between uncomplicated malaria and severe malaria [28] with a sensitivity of 86% (95% CI 71–94) and a specificity of 85% (95% CI 76–92) (see Additional file 7). As expected, the more the disease severity states reflected both ends of the spectrum (e.g., uncomplicated malaria versus cerebral malaria) the better they could be distinguished [25, 28, 29, 32] (see Additional file 7).

Since cerebral malaria is a complication of falciparum malaria and therefore a manifestation of severe malaria, distinguishing cerebral malaria from other forms of severe malaria was difficult, as shown by the observation that only the Ang-1 concentration had a predictive value significantly higher than chance [28] with a sensitivity of 72% (95% CI 56–84) and specificity of 66% (95% CI 55–75). The predictive value of the Ang-2 concentration for fatal outcome was found to be as well as plasma lactate in Vietnamese adults with severe malaria [33] and in Malawian children with cerebral malaria [27], and even better in Indonesian adults with severe malaria [34] (see Additional file 7). In order to increase the discriminative power, two studies used a combination of markers [27, 30]. Incorporating the Ang-2 concentration or the Ang-1, Ang-2 and Tie-2 concentrations in a clinical model, comprising age, body condition score, respiratory distress, and severe anaemia as other parameters, significantly improved the ability to accurately predict survival or fatal outcome compared to the clinical model alone (c-index (95% CI), p value) (*i*: 0.78 (0.68–0.82), 0.02; *ii*: 0.79 (0.72–0.84), 0.03) [27]. The combination of Ang-2 with CXCL10 and sICAM-1 had a sensitivity of 100% and a specificity of 93% to distinguish survivors from non-survivors) [30].

In addition to the use as biomarker for disease severity in humans infected with *Plasmodium* species, the Ang-1 and Ang-2 concentration may also be used for distinguishing cerebral malaria from other diseases involving the central nervous system (CNS) in critically ill patients. Distinguishing cerebral malaria from other febrile diseases affecting the CNS is challenging, but can have major treatment implications. Ang-1 was found to differentiate between cerebral malaria, diagnosed by malaria specific retinopathy, and other febrile CNS-affecting diseases with a sensitivity and specificity of 88% (95% CI 69–96) and 87% (95% CI 73–94), respectively (see Additional file 7) [29].

#### Ang-1 and Ang-2 levels do not correlate with parasitaemia, sequestration and erythrocyte rosetting

Assuming that Ang-1 and Ang-2 concentrations and parasitaemia, parasite biomass, sequestration, and erythrocyte rosetting all reflect disease severity during malaria, a tight correlation between them may be anticipated. However, no correlation was found between concentrations of Ang-1 and Ang-2 or Ang-2/Ang-1 ratio and sequestration in cerebral malaria in brain autopsies [33], microvascular obstruction measured with an Orthogonal Polarization Spectral device in the rectal mucosa [46] or rosetting as determined by IgG antibody levels to the infected erythrocyte surface of ex vivo isolates and corrected for disease severity [49]. Parasite biomass, as measured by *Pf*HRP2, was correlated with Ang-2 in *P. falciparum* [34, 43, 46], but not as measured by PvLDH and pLDH in *P. vivax.* [43]. Three studies could not find a correlation between parasitaemia and Ang-1 and Ang-2 in *P. falciparum*-infected children and adults [25, 33, 34]. However, three studies did observe a correlation between Ang-2 and the parasitaemia in *P. falciparum*- and *P. vivax*-infected children and adults [28, 43] or the Ang-2/Ang-1 ratio [33]. Taken together, the relation between Ang-1 and Ang-2 and parasite abundance-related markers varies between the studies.

### Aim II: Adjuvant therapy next to anti-malarial treatment

#### Ang-1 and Ang-2 levels are influenced by anti-inflammatory therapy

Ang-1 and Ang-2 concentrations have both been suggested as adjuvant treatment targets in malaria [15, 53], but so far interventional studies in mice models or in humans have not been performed. Several studies used Ang-1 and Ang-2 as biomarkers for endothelial cell activation after an intervention aiming at another target.

#### Adjuvant treatment strategies

In distinct studies, *P. berghei* (Anka strain*)*-infected mice and/or *P. falciparum*-infected humans received next to artesunate either a functional S1P receptor antagonist (FTY720 or LX2031 [38], a PPAR-γ agonist: rosiglitazone [26] or inhaled nitric oxide [40–42]. FTY720 and LX2931 influence the homeostasis of inflammation and of vascular endothelium activity [54–56]. Rosiglitazone increases CD36-mediated phagocytosis of infected erythrocytes, has anti-inflammatory properties and improved survival in a cerebral malaria model previously [57, 58]. NO inhibits the exocytosis of Weibel–Palade bodies [59], organelles in endothelial cells that store both vWF and Ang-2. NO levels and cofactors required for NO synthesis are known to be reduced during *P. falciparum* infection [34, 35, 44] and higher NO levels are associated with lower Ang-2 levels [34], whereas NO inhibitors positively correlate with Ang-2 [45]. Several processes contributing to the decreased NO levels during malaria have been studied, such as substrate limitation and increase in NO inhibitors [35], but these are beyond the scope of this review.

#### Adjuvant treatment in mice

No change in Ang-1 levels have been observed in mice treated with FTY720, one, three or five days after infection nor an increase in survival in treated mice three or five days after infection [38]. The survival rates also did not increase in both prophylactic and post-infection LX2931-treated mice. However, a higher Ang-1 concentration and survival rate were observed in mice receiving prophylactic treatment with either FTY720 or iNO compared to placebo-treated mice [38] (see Additional file 6). Survival rates increased in mice receiving adjuvant treatment with rosiglitazone or iNO three or five days after infection compared to artesunate-only treated mice [40]. Rosiglitazone prevented the decrease in the Ang-1 concentration that was seen in mice treated only with artesunate [26]. The Ang-2/Ang-1 mRNA levels, measured in homogenized brain tissue of mice, were significantly lower in mice prophylactically treated with iNO [40] or post-infectiously with rosiglitazone compared to mice treated only with artesunate [26]. These results demonstrated an association between prophylactic therapy, decreased endothelium activation and increased survival rates.

When rosiglitazone was given to *P. berghei*-*Anka*-infected mice with one Ang-1 allele deleted (Ang-1^del^), production of the Ang-1 levels was 30–50% compared to wild type mice five to six days post-infection [26]. An increase in survival was only seen in wild type mice treated with rosiglitazone and not in the Ang-1^del^ mice treated with rosiglitazone [26]. This emphasizes that proper Ang-1 production apparently increases survival in a direct or indirect way, and therefore, a role of Ang-1 in the pathogenesis during malaria is likely.

#### Adjuvant treatment in humans

iNO as adjuvant therapy for severe malaria and rosiglitazone for uncomplicated malaria was also studied in humans. In a total of 272 Ugandan children, adjuvant iNO was given in two studies with either nitrogen [42] or air [41] as placebo treatment. No significant differences in Ang-1, Ang-2 concentrations nor survival between the iNO-treated and placebo groups was found in either study. In rosiglitazone-treated patients the Ang-2/Ang-1 ratio was significantly lower compared to patients receiving placebo, as measured three days after treatment was started and parasite clearance was significantly enhanced [26]. None of the patients died or was admitted to intensive care in both the treatment and control groups.

## Discussion

The role of Ang-1 and Ang-2 as biomarkers and possible therapeutic targets in severe malaria was evaluated in this systematic review. Decreased Ang-1 and increased Ang-2 concentrations seem to be valuable as biomarker for disease severity and survival. The role of Ang-1 and Ang-2 in follow-up of treatment effectiveness, as well as their role as future therapeutic targets, are promising, but further studies are warranted. Nonetheless, some discussion points before use as biomarkers or therapeutic targets remain.

### Ang-1 and Ang-2 as biomarkers for disease severity

#### Uncomplicated malaria *versus* severe malaria and cerebral malaria

The results indicate that the decreased Ang-1 and the increased Ang-2/Ang-1 ratio are robust biomarkers to distinguish uncomplicated malaria from cerebral malaria [25, 28, 29, 32]. However, it is easier to distinguish uncomplicated malaria from cerebral malaria by a rapid clinical examination procedure as defined by WHO (cerebral malaria: severe *P. falciparum* with coma (Glasgow coma scale <11, Blantyre coma score <3), or malaria with coma persisting >30 min after a seizure). Nevertheless, Ang-1, Ang-2 and the Ang-2/Ang-1 ratio could be valuable markers if they can identify patients with uncomplicated malaria at risk for progressing to severe disease. The area under the receiver operating characteristic (AUROC) for distinguishing uncomplicated malaria from severe malaria is 0.88 (p = 0.001) and the sensitivity and specificity are 86% (95% CI 71–94) and 85% (95% CI 77–92), respectively [28] (see Additional file 7). This AUROC is higher than for plasma lactate [0.67 (95 CI % 0.57–0.75)] or parasitaemia [0.53 (95% CI 0.45–0.62] as found in an endemic area [60] and the sensitivity higher than in non-immune travellers (67%) [61]. Although the discriminating power of Ang-1 levels is promising, conclusion should be drawn with caution, as only one study in children (N = 193) evaluated the AUROC to distinguish uncomplicated malaria from severe malaria [28].

#### Survivors versus non-survivors

Ang-1 and Ang-2 levels can be used to distinguish malaria disease severity states but prediction of outcome might be of more clinical value. Several studies that focussed on clinical outcome demonstrated that the Ang-2 level predicted mortality as well as, or better than, lactate blood levels in adults and children in Asia and Africa with severe and cerebral malaria [27, 33, 34]. Additional prospective studies that evaluate Ang-1 and/or Ang-2 as markers of disease progression and clinical outcome should be performed to confirm these results.

#### Cerebral malaria versus other febrile CNS-affecting diseases

A marker other than parasitaemia to distinguish cerebral malaria from ‘other diseases with involvement of the CNS’ might be useful in patients with parasitaemia, as asymptomatic mild parasitaemia is not uncommon in malaria-endemic areas [62, 63]. Direct and indirect ophthalmoscopy can be used for this if retinopathy is observed with certain malaria-specific characteristics, such as retinal whitening, vessel changes, retinal haemorrhages, and papilledema. This clinical procedure has a sensitivity of 95% and a specificity of 90% for diagnosing cerebral malaria [64]. The Ang-1 concentration and the Ang-2/Ang-1 ratio were found to be good discriminators between ‘cerebral malaria with retinopathy’ and ‘other diseases with involvement of the CNS’ (see Additional file 7). However, cerebral malaria with retinopathy is already a selected group within the total group of patients with cerebral malaria, and therefore, it might overestimate the sensitivity and specificity to diagnose cerebral malaria. In addition, the group of patients that will benefit from a marker for cerebral malaria (patients with mild parasitaemia and a non-malaria disease that involves the CNS), were not represented in both studies that investigated the capacity of angiopoetin levels to distinguish cerebral malaria from other diseases with involvement of the CNS as parasitaemia in the latter group was absent. Nevertheless, to evaluate whether Ang-1 and/or Ang-2 levels can identify these patients might be of clinical value.

### Treatment follow-up

Early parameters that indicate whether treatment is effective in malaria patients are valuable as treatment regimens may be reconsidered when patients do not properly recover. In both humans [26, 29, 34, 36, 37, 42] and mice [26, 38–40] recovery from malaria and survival was associated with an increase in Ang-1 and decrease in Ang-2 level (see Additional files 3, 4, 5, 7). However, the threshold for an Ang-1 increase or Ang-2 decrease that reflects proper recovery was not investigated. Neither were Ang-1 and Ang-2 levels compared with other clinical or laboratory indices, e.g., fever or parasite clearance, which would be of interest.

### Variation between studies

Even though Ang-1 and Ang-2 concentrations could discriminate between disease severity states during malaria, distinct studies found varying levels of sensitivity and specificity (see Additional file 7). This variation may be due to: (1) host-related factors, such as genetic differences between study populations and differences in acquired immunity towards *Plasmodium* species infection between study populations; (2) pathogen-related factors, such as infections with different strains with inherently differences in pathogenicity; and, (3) environment-related factors, such as differences in access to healthcare that may result in delayed diagnosis or delayed start of proper treatment. In addition, the methods by which Ang-1 and Ang-2 levels are determined are further developing and currently lack proper standardization. Additional investigations are required before conclusions on the varying levels can be drawn.

### Ang-1 and platelet activation

A lower Ang-1 level was found in healthy controls compared to *P. falciparum*-infected patients in one study which collected blood in CTAD tubes [36]. These tubes contain the platelet-stabilizing agents citrate, theophylline, adenosine, and dipyridamole, which together result in a nearly complete block of in vitro platelet activation [65, 66]. The studies determining Ang-1 in serum [32] or in venous blood (not further specified) [25] found a higher level of Ang-1 in healthy controls compared to *P. falciparum*-infected patients. In addition, other studies that determined Ang-1 in malaria patients found a negative correlation between Ang-1 and disease severity. It is remarkable that the only study using tubes that avoid in vitro platelet activation found a higher level of Ang-1, which is stored in platelets and released upon activation, in patients than in healthy controls. Platelet activation may occur in vivo during malaria as induction of coagulation is proven even in early malaria [67]. To exclude altered Ang-1 levels due to in vitro platelet activation after blood collection, platelet-poor specimens are preferred to determine platelet activation in vivo. 11-dehydrothromboxane B2 is a breakdown product of thromboxane A2 that is produced in vivo by activated platelets. Since 11-dehydrothromboxane B2 is excreted in urine, a specimen that is free of platelets, determination of 11-dehydrothromboxane B2 in urine samples may estimate in vivo platelet activation most accurately [68].

### Lack of consistent associations between Ang-1, Ang-2 and the parasitaemia in *Plasmodium falciparum*

Even though Ang-1 and Ang-2 concentrations correlate with disease severity, the results of the correlation between Ang-1 or Ang-2 concentrations in *P. falciparum* infection and parasitaemia, sequestration or rosetting vary between the studies [25, 28, 33, 34, 43, 46, 49]. The lack of a consistent correlation of the Ang-1 concentration, Ang-2 concentration and Ang-2/Ang-1 ratio with parasitaemia may be due to the fact that this parameter is determined in peripheral blood and does not reflect the true parasite load as sequestered parasites are not adequately taken into account. A correlation between *Pf*HRP2, which reflects total parasite load during *P. falciparum* infection and Ang-2 was found in three studies [34, 43, 46], but sequestration [46] and rosetting corrected for disease severity [49] did not correlate with Ang-1 and/or Ang-2 concentrations. The correlation between Ang-2 with parasite load but not with sequestration and rosetting might be due to: (1) the fact that *Pf*HRP2 levels [34, 43, 46], measuring sequestration with a OPD device [46] and IgG antibody levels [49] are all markers that may not estimate their biological phenomena similarly; (2) a temporal dissociation of the determinations [46]; (3) loss of correlation due to the fact that locally observed phenomena (sequestration and rosetting) were compared to a systemic phenomenon (Ang-2 release) [46]; or, (4) the infected erythrocytes may activate endothelial cells not by direct, physical contact, but indirectly by inducing other endothelial cell activators, such as cytokines [69, 70]. Next to the quantity of parasite biomass, the *Plasmodium* strain and the host response might contribute to the variations in Ang-2 increase, although it should be noted that an included study [49] did not show a relation between Ang-2 and group A-like *Pf*EMP expression if corrected for disease severity. However, the possible differences in other genes were not examined in that study. Taken together, the host-response-related marker Ang-2 might be a good or better biomarker for disease severity and treatment follow-up than pathogen-related markers, such as parasite load or parasitaemia.

### Ang-2 in vivax *versus* falciparum malaria

Data on Ang-2 levels in *P. vivax* and *P. falciparum* differed between distinct studies: two studies found a higher Ang-2 level in *P. falciparum* compared to *P. vivax* [43, 47] and one study found a higher Ang-2 level in *P. vivax* compared to *P. falciparum* [48]. However, comparison should be done with caution as these studies were performed in different populations: patients in endemic areas [43, 47] versus travellers in non-endemic regions [48] and the parasitaemia was not reported in all studies [48]. In addition, the blood samples of *P. vivax* and *P. falciparum* of infected Canadian travellers were processed differently [48], which may have influenced the Ang-2 levels results. Higher Ang-2 levels in *P. vivax* compared to *P. falciparum* are plausible as *P. vivax* is known to induce a stronger cytokine response [4], which was also shown in an included study in adults in an endemic area [47]. These cytokines might contribute to endothelial cell activation and therefore to Ang-2 release from Weibel–Palade bodies. Interestingly, in *P. vivax*-infected patients Ang-2 levels did not correlate with pLDH and PvLDH, which were proposed as markers that take both peripheral and hidden parasite load into account. Instead, a correlation was found between Ang-2 levels and parasitaemia. This might suggest a cytokine-independent endothelial cell activation, as both hidden and peripheral-infected erythrocytes should contribute to the induction of the cytokine response. On the other hand, pLDH and PvLDH are not as well-established markers for the parasite biomass in *P. vivax*-infected patients [71] as *Pf*HRP2 is in *P. falciparum*-infected patients, and therefore, no firm conclusions can be drawn yet. Additional research is necessary to elucidate the differences in pathogenesis between vivax and falciparum malaria and the role of endothelium activation during both *Plasmodium* infections.

### Clinical implementation

Although Ang-1 and Ang-2 levels can be used to differentiate between disease severity states in malaria disease and might predict disease progression, their use in routine clinical practice is not yet possible. Malaria disease mainly occurs in resource-poor settings where it is not possible to determine Ang-1 or Ang-2 with complex laboratories techniques. If Ang-1 and Ang-2 are shown to be robust biomarkers for disease progression, new simple rapid-point-of-care tests need to be developed for clinical use, as was done before for CRP [72] and for multiple haematological, biochemical and coagulation parameters [73]. Furthermore, the clinical consequences of a marker for disease progression are currently limited as no adjuvant treatment is yet available, next to medication, to eradicate the parasite. However, development of adjuvant treatment and biomarkers to indicate which patients should receive adjuvant treatment go hand-in-hand and multiple adjuvant treatment strategies are currently investigated (as reviewed by [74, 75]).

### The possible role of Ang-1 and Ang-2 in adjuvant treatment regimens for falciparum malaria

Studies using Ang-1 or Ang-2 as therapeutic target were only performed in sepsis, which shares many similarities with severe malaria caused by a *P. falciparum* infection. Both sepsis and malaria are characterized by cytokines release, coagulation alterations and endothelial cell activation [15, 18, 23]. In sepsis, Ang-1 was the mostly studied angiopoietin in interventional murine models (as reviewed by [24]). Ang-1 was found to be protective in endotoxin-induced shock or hyperoxia-induced lung injury models. However, most studies used Ang-1 treatment prior to infection, which is not useful in a clinical setting for sepsis nor for malaria. Some of the studies giving Ang-1 after development of sepsis or acute lung injury found no reduction in mortality while others reported a positive effect [24]. In addition, it should be noted that *P. berghei* ANKA-infected CB57BL/6 mice, which are widely used as a model for human cerebral malaria, do show histopathological features that differ substantially from cerebral malaria in humans. Human cerebral malaria is characterized by sequestration, whereas inflammation is central in murine cerebral malaria and the amount of sequestration limited [76]. These differences may contribute to the contrasting results in outcome between iNO-treated, *P. berghei* ANKA-infected mice and *P. falciparum*-infected humans. This is supported by the observation that most successful interventions in murine cerebral malaria show no significant effect in human cerebral malaria (as reviewed by [77]). More investigations on the therapeutic effect of post-infection use of Ang-1 in different disease models are needed before solid conclusions about the therapeutic properties of Ang-1 and/or Ang-2 can be drawn.

**Fig. 2:**
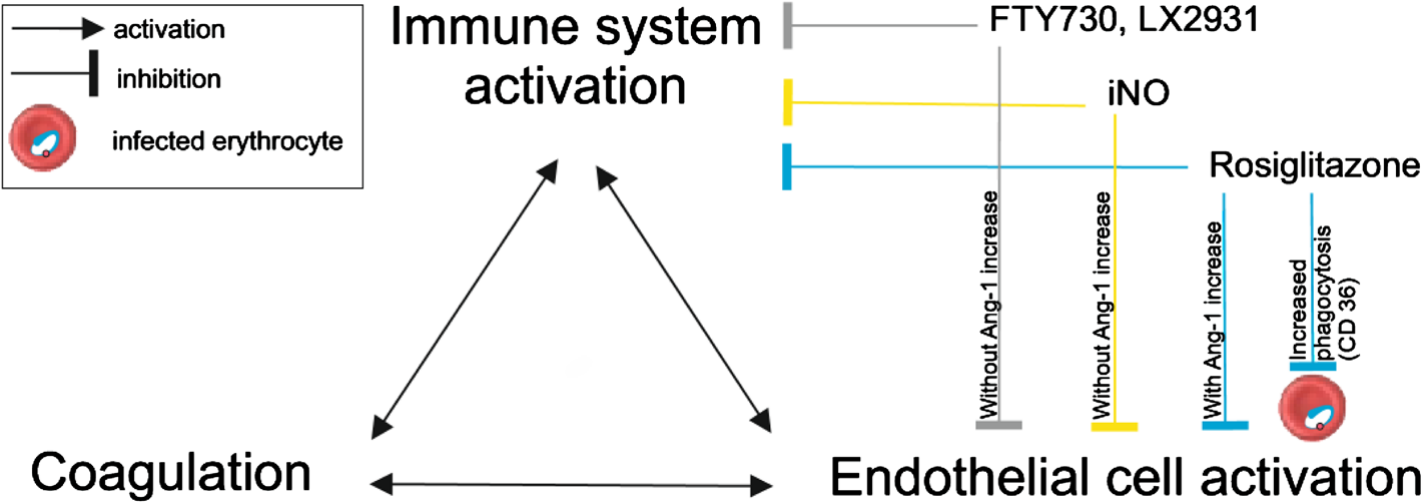
Interactions between different regulatory systems and the effects of therapeutics on the determinants. Coagulation, endothelial cell activation and immune system activation are processes that stimulate each other. FTY730, LX2931 and NO inhibit the immune system activation and endothelial cell activation. Rosiglitazone inhibits immune system activation, endothelial cell activation and upregulates CD36, which results in increased phagocytosis of the infected erythrocytes

The complexity of the host response to *Plasmodium* species infections possibly plays a role in the lack of effect of adjuvant treatment when given post infection. During malaria disease in humans and mice the immune system, coagulation system and the activity of endothelial cell are all systematically altered. These processes are complex and interweaved with each other (see Fig. 2). Providing even multi-targeted adjuvant treatment with FTY720 or iNO at the moment that these regulatory systems are already systemically activated and upregulating each other may have too limited an effect to halt these feed-forward stimulated processes. Multi-targeted adjuvant treatment, such as rosiglitazone, that (unlike FTY720 or iNO) was found to have an effect on Ang-1 and Ang-2 levels and survival when administered post-infection, may however interfere with all the distinct regulatory systems in a way that reduces morbidity and mortality [26] (see Fig. 2), although further studies are definitely needed.

## Conclusion

Ang-1 and Ang-2 concentrations correlate with disease severity and survival in malaria and show a good discriminative power to distinguish disease severity states with predictive values equal to, or higher than, the currently used biomarkers plasma lactate and parasitaemia. However, different disease severity states were compared in the distinct studies and not every distinction has clinical impact. The possible ability of Ang-1 and Ang-2 concentrations to distinguish cerebral malaria from other febrile CNS-affecting diseases and to predict those patients progressing from uncomplicated malaria towards severe malaria will have clinical value and should be studied in more detail before it can be used in routine practice.

For further optimization of treatment of patients with severe disease, multi-targeted adjuvant treatment regimens are needed. Agents interfering with Ang-1 and or Ang-2 may be included in such adjuvant therapy as their role as therapeutic target is promising. However, it is conceivable that agents interfering with multiple pathways are necessary, since multiple regulatory systems are consecutively activated during malaria infection.

## Additional files

**Additional file 1.** Search strategy.

**Additional file 2.** Flow diagram: selection of relevant articles.

**Additional file 3.** Studies on children and adults showing significant differences in Ang-1 and Ang-2 levels in *Plasmodium falciparum* and *Plasmodium vivax* infection.

**Additional file 4.** Studies on adults showing significant differences in Ang-1 and Ang-2 levels in *Plasmodium falciparum* and *Plasmodium vivax* infection.

**Additional file 5.** Studies on children showing significant differences in Ang-1 and Ang-2 levels in *Plasmodium falciparum* infection.

**Additional file 6.** Studies on the effect of anti-inflammatory interventions on Ang-1 and Ang-2 levels during *Plasmodium spp.* infections in humans and mice.

**Additional file 7.** Ang-1 and Ang-2 as biomarkers for disease severity during human *Plasmodium spp.* infection.

## Abbreviations

Ang-1/-2: angiopoietin-1/angiopoietin-2
AUROC: area under the receiver operating characteristic
CNS: central nervous system
CRP: C-reactive protein
(i)NO: (Inhaled) nitric oxide
*Pf*EMP-1: *Plasmodium falciparum* erythrocyte membrane protein-1
P(v)LDH: *Plasmodium (vivax*) lactate dehydrogenase
vWF: von Willebrand factor
WPB: Weibel–Palade bodies
