# Supplementary material for: Systematic review of the role of angiopoietin-1 and angiopoietin-2 in Plasmodium species infections: biomarkers or therapeutic targets?
Source: Malar J. 2016 Dec 1;15:581. doi: 10.1186/s12936-016-1624-8 (PMC5134107; doi:10.1186/s12936-016-1624-8)
Supplement: Supplementary file 1 — Additional file 1. Search strategy. [file 12936_2016_1624_MOESM1_ESM.docx]

**Additional file 1 – Search strategy**

Search performed on 15 June 2016

| Database | Number of publications | Number of publications after deduplication |
| --- | --- | --- |
| Embase.com | 170 | 166 |
| Medline (ovid) | 45 | 9 |
| Web-of-science | 112 | 32 |
| Scopus | 128 | 2 |
| Cochrane | 5 | 0 |
| cinahl (ebsco) | 5 | 1 |
| lilacs | 3 | 2 |
| scielo | 3 | 0 |
| ProQuest | 8 | 8 |
| Google scholar | 100 | 58 |
| **Total** | **579** | **278** |

Table 1: Number of publications extracted from the indicated databases**.**

**Syntaxis of search strategies**

**Embase.com**

(angiopoietin/exp OR 'angiopoietin 1'/exp OR 'angiopoietin 2'/exp OR 'angiopoietin receptor'/exp OR 'angiogenic protein'/exp OR 'angiogenic factor'/exp OR (angiopoietin* OR angiogenic* OR angpt* OR 'ang 1' OR 'ang 2' OR ang1 OR ang2 OR (tie NEAR/3 (receptor* OR protein*)) OR tek OR tie1 OR tie2 OR (Tyrosine* NEAR/3 kinase* NEAR/3 immunoglobulin*))) AND (malaria/exp OR Plasmodium/exp OR 'antimalarial agent'/de OR (malaria OR Plasmodium OR antimalari*))

**Medline (ovid)**

(exp angiopoietins/ OR exp "Receptors, TIE"/ OR "Angiogenic Proteins"/ OR (angiopoietin* OR angpt* OR "ang 1" OR "ang 2" OR ang1 OR ang2 OR (tie ADJ3 (receptor* OR protein*)) OR tek OR tie1 OR tie2 OR (Tyrosine* ADJ3 kinase* ADJ3 immunoglobulin*))) AND (exp malaria/ OR exp Plasmodium/ OR "Antimalarials"/ OR (malaria OR Plasmodium OR antimalari*))

**Cochrane**

((angiopoietin* OR angiogenic* OR angpt* OR 'ang 1' OR 'ang 2' OR ang1 OR ang2 OR (tie NEAR/3 (receptor* OR protein*)) OR tek OR tie1 OR tie2 OR (Tyrosine* NEAR/3 kinase* NEAR/3 immunoglobulin*))) AND ((malaria OR Plasmodium OR antimalari*))

**Web-of-science**

TS=(((angiopoietin* OR angiogenic* OR angpt* OR "ang 1" OR "ang 2" OR ang1 OR ang2 OR (tie NEAR/3 (receptor* OR protein*)) OR tek OR tie1 OR tie2 OR (Tyrosine* NEAR/3 kinase* NEAR/3 immunoglobulin*))) AND ((malaria OR Plasmodium OR antimalari*)))

**Scopus**

TITLE-ABS-KEY(((angiopoietin* OR angiogenic* OR angpt* OR "ang 1" OR "ang 2" OR ang1 OR ang2 OR (tie W/3 (receptor* OR protein*)) OR tek OR tie1 OR tie2 OR (Tyrosine* W/3 kinase* W/3 immunoglobulin*))) AND ((malaria OR Plasmodium OR antimalari*)))

**cinahl (ebsco)**

(MH "Angiogenic Proteins" OR (angiopoietin* OR angpt* OR "ang 1" OR "ang 2" OR ang1 OR ang2 OR (tie N3 (receptor* OR protein*)) OR tek OR tie1 OR tie2 OR (Tyrosine* N3 kinase* N3 immunoglobulin*))) AND (MH malaria+ OR MH "Antimalarials" OR (malaria OR Plasmodium OR antimalari*))

**Google scholar**

angiopoietin|angiopoietins|angiogenic|angpt|angpt1|angpt2|angpt3|angpt4|"ang|tie 1|2|3|4"|ang1|ang2|ang3|ang4|"tek|tie1|tie2|tie receptor|protein"|"Tyrosine kinase immunoglobulin" malaria|Plasmodium|antimalaria

**lilacs**

**scielo**

(angiopoietin* OR angiogenic OR angpt* OR ang1 OR ang2 OR ang3 OR ang4 OR tie1 OR tie2 OR "tie receptor" OR "tie protein" OR "Tyrosine kinase immunoglobulin") AND (malaria OR Plasmodium OR antimalaria*)

**ProQuest**

(ti(angiopoietin* OR angiogenic OR angpt* OR ang1 OR ang2 OR ang3 OR ang4 OR tie1 OR tie2 OR "tie receptor" OR "tie protein" OR "Tyrosine kinase immunoglobulin") OR ab(angiopoietin* OR angiogenic OR angpt* OR ang OR tie OR ang1 OR ang2 OR ang3 OR ang4 OR tie1 OR tie2 OR "tie receptor" OR "tie protein" OR "Tyrosine kinase immunoglobulin")) AND (ti(malaria OR Plasmodium OR antimalaria*) OR ab(malaria OR Plasmodium OR antimalaria*))
