# Supplementary material for: Systematic review of the role of angiopoietin-1 and angiopoietin-2 in Plasmodium species infections: biomarkers or therapeutic targets?
Source: Malar J. 2016 Dec 1;15:581. doi: 10.1186/s12936-016-1624-8 (PMC5134107; doi:10.1186/s12936-016-1624-8)
Supplement: Supplementary file 2 — Additional file 2. Flow diagram: selection of relevant articles. [file 12936_2016_1624_MOESM2_ESM.doc]

**Additional file 2 – Flow diagram: selection of relevant articles**

**Screening**

**Included**

**Eligibility**

**Identification**

Records identified through database searching
(n = 579)

Additional records identified through other sources (references of articles) (n=0)

(n = 0)

Records after deduplication
(n = 278)

Records screened
(n = 278)

Records excluded (title/abstract)
(n = 192)

Full-text articles assessed for eligibility
(n = 86)

Full-text articles excluded, with reasons. (n = 60)

- Not an article (n= 15)
- (systematic) review (n=14)
- No determination of angiopoietin level (n=25)
- Pregnancy related (n=6)

Studies included in qualitative synthesis
(n = 26)

Studies included in quantitative synthesis (meta-analysis)
(n = 0)
