# Supplementary material for: Systematic review of the role of angiopoietin-1 and angiopoietin-2 in Plasmodium species infections: biomarkers or therapeutic targets?
Source: Malar J. 2016 Dec 1;15:581. doi: 10.1186/s12936-016-1624-8 (PMC5134107; doi:10.1186/s12936-016-1624-8)
Supplement: Supplementary file 3 — Additional file 3. Studies on children and adults showing significant differences in Ang-1 and Ang-2 levels in Plasmodium falciparum and Plasmodium vivax infection. [file 12936_2016_1624_MOESM3_ESM.docx]

|  | **References** | **Population, N**  **Study type**  **Age in years** | **Ang-1 (ng/ml)** | **Ang-2 (ng/ml)** | **Ang2/Ang1 ratio** |
| --- | --- | --- | --- | --- | --- |
| *Pf.* | Conroy et al. (2009) | Thailand, N=193  Prospective cohort study  UM: 26 (13-50)  SM: 24 (14-59)  CM: 26 (14-61) | UM > CM > SM | UM < SM and CM | UM < SM and CM |
|  | Jain et al. (2011) | India, N=183  Retrospective cohort study  HC: 25 (14-32)  MM: 19 (12-36)  CM-S: 25 (12-40)  CM-NS: 25 (13.5-37.5) | HC (12.4) > MM (4.6) > CM S (2.8) and CM NS (2.4) | HC (0.34) < MM (0.97) < CM S (3.6) < CM NS (7.8) | HC (0.02) < MM (0.16) < CM S (1.42) < CM NS (2.69) |
|  | Brouwers et al. (2015) | Indonesia, N=52  Prospective case-control study  HC: 24 (22-29)  Malaria:13 (6-26) | HC < malaria  Further increase after start treatment | HC < malaria  Decline after start treatment | HC = malaria |
|  | MacMullin et al. (2012) | Canada, N=186  Retrospective cohort study  0-55+ | FB = CB | FB = CB;  *Pv.* (0.34) < *Pf.* (1.1)*  FB: *Pv.* (0.31) < *Pf.* (1.0)*  CB: *Pv*=*Pf* |  |
| *Pv.*/*Pf* | Barber et al. (2015) | Malaysia, N=266  Prospective cohort study  Controls: 35 (23-44)  Non-severe *Pv*.:24 (18-29)  Severe *Pv*.: 39 (30-52)  Non-severe *Pf*.: 25 (17-39) Severe *Pf*.: 33 (19-45) | Not determined | HC (1.2) < non-severe *Pv.* (4.6) < severe *Pv.* (8.9)  non severe *Pf.* (3.2) < severe *Pf.* (8.4) |  |

**Additional file 3 – Studies on children and adults showing significant differences in Ang-1 and Ang-2 levels in *Plasmodium falciparum* and *Plasmodium vivax* infection.**

Variables were presented as median; inter quartile range (IQR) unless otherwise specified; **CB**, Canadian born; **CM,** cerebral malaria (WHO definition); **FB**, foreign born; **HC**, healthy control; **MM**, Mild malaria, defined as parasitemia of < 25,000 parasites/μl and no evidence of severe malaria and no past history of mental/metabolic illness, tuberculosis, meningitis, or accidental head injury; ***Pf.***, *Plasmodium falciparum;* ***Pv.***, *Plasmodium vivax*; **S**, survivors, **SM**, severe malaria; **UM**, uncomplicated malaria.

* Mean
