# Supplementary material for: Systematic review of the role of angiopoietin-1 and angiopoietin-2 in Plasmodium species infections: biomarkers or therapeutic targets?
Source: Malar J. 2016 Dec 1;15:581. doi: 10.1186/s12936-016-1624-8 (PMC5134107; doi:10.1186/s12936-016-1624-8)
Supplement: Supplementary file 4 — Additional file 4. Studies on adults showing significant differences in Ang-1 and Ang-2 levels in Plasmodium falciparum and Plasmodium vivax infection. [file 12936_2016_1624_MOESM4_ESM.docx]

**Additional file 4 – Studies on adults showing significant differences in Ang-1 and Ang-2 levels in *Plasmodium falciparum* and *Plasmodium vivax* infection.**

|  | **References** | **Population, N**  **Study type**  **Age in years** | **Ang-1 (ng/ml)** | **Ang-2 (ng/ml)** | **Ang2/Ang1 ratio** |
| --- | --- | --- | --- | --- | --- |
| *Pf.* | Yeo et al. (2008, 2010 (ref. 35)) | Indonesia, N=146  Prospective cohort study  HC: 25 (18–44)**  MSM: 28 (18–56)**  SM: 29 (18–56)** | Not determined | HC (2.3) < MSM (5.3) < SM (15.0)*  SM S < SM NS (admission levels)  Decrease in SM S (2.7 ng/ml/24h) during treatment but not in SM NS |  |
|  | Lovegrove et al. (2009) | Thailand, N=60  Prospective cohort study  HC: 32 (25-48)*  UM: 22 (14–63*  CM: 25 (17–50)* | HC (378) > UM (82.25) > CM (3.51)* | HC (0.0089) < UM (1.84) < CM (6.19)* | HC (3*10^-5^) < UM (0.017) < CM (3.47)* |
|  | Prapansilp et al. (2013) | Vietnam, N=63 SM, CM patients, 23 were used for autopsia  UK, N=18 autopsy controls  Retrospective case-control study  Controls: 58.5 (36-74.5)*  Cases: 32 (26-43)* | Lower incidence of high expression in neurons/astroglial cells in non-malaria vs SM and CM, not in vessels. | Lower incidence of high expression in neurons and lower expression in astroglial cells in non-malaria vs SM and CM.  S (15) < NS (28)* | S (30.6) < NS (42.5)* |
|  | Hanson et al. (2015) | Bangladesh, India, N=142  Prospective observational study  35 (25–45)* | Not determined | HC (1.6) < SM (19.1)*  S (14.9) < NS (21.9)*  comatose patients < not comatose patients |  |
| *Pv.*/*Pf* | Yeo et al. (2010) (ref. 48) | Indonesia, N=143  Prospective observational study  HC: 25 (18–44)**  *Pf*.28 (18–56)**  *Pv.* 23 (18–35)** | Not determined | HC (2.3) *<*uncomplicated *Pf.* (5.3) *<* uncomplicated *Pv.* (7)* |  |
| *Pv.* | Gomes et al. (2015) | Brazil, N=80  Retrospective case-control study  Case: 38.5 (25-55)*  Control 39 (28-49)* | Non-severe (19.9) > severe (12)*  Normal platelet count > thrombocytopenia | Non-severe (5.9) < severe (8.2)*  Normal platelet count < thrombocytopenia | Non-severe (0.3) < severe (0.8)*  Normal platelet count < thrombocytopenia |

**CM,** cerebral malaria (WHO definition); **HC**, healthy control; **MSM**, moderate severe malaria, defined as fever within the preceding 48 h, with >1,000 asexual *P. falciparum* parasites/L, with no WHO warning signs or criteria for SM; **NS**, non-survivors; ***Pf.***, *Plasmodium falciparum;* ***Pv.***, *Plasmodium vivax*; **S**, survivors, **SM**, severe malaria; **UM**, uncomplicated malaria.

*Median (IQR), **Mean (range)
