# Supplementary material for: Systematic review of the role of angiopoietin-1 and angiopoietin-2 in Plasmodium species infections: biomarkers or therapeutic targets?
Source: Malar J. 2016 Dec 1;15:581. doi: 10.1186/s12936-016-1624-8 (PMC5134107; doi:10.1186/s12936-016-1624-8)
Supplement: Supplementary file 5 — Additional file 5. Studies on children showing significant differences in Ang-1 and Ang-2 levels in Plasmodium falciparum infection. [file 12936_2016_1624_MOESM5_ESM.docx]

**Additional file 5 - Studies on children showing significant differences in Ang-1 and Ang-2 levels in *Plasmodium falciparum* infection.**

| **References** | **Population; N**  **Study**  **Age in years** | **Ang-1 (ng/ml)** | **Ang-2 (ng/ml)** | **Ang2/Ang1 ratio** |
| --- | --- | --- | --- | --- |
| Lovegrove et al. (2009) | Uganda, N=164  Prospective cohort study  HC: 7 (3.2–12)*  UM: 7 (3.0–12)*  CM: 5.4 (3.2–12)* | HC (64.4) > UM (25.0) > CM (9.0)*  CM S (9.1) > CM NS (0.39) | HC (0.068) < UM (0.28) < CM (0.83) * | HC (0.0015) < UM (0.013) < CM (0.14)*  CM S (0.13) < CM NS (2.6) |
| Conroy et al. (2010) | Malawi, N=123  Retrospective case-control study  UM: 3.3 (0.7-8.0)*  NMC: 2.2 (0.1-9.1)*  CM-N: 4.3 (1.2-13.3)*  CM-R: 2.8 (1.1-6.8)* | UM and NMC > CM-R  CM-N > CM-R  Admission level CM-R survivors < 28 days post-treatment | UM and NMC < CM-R  CM-N < CM-R  Admission level CM-R > 28 days post-treatment | UM and NMC < CM-R  CM-N < CM-R  Admission level CM-R > 28 days post-treatment |
| Erdman et al. (2011) | Uganda, N=156  Retrospective case-control study  UM: 4.4 (2.1-8.1)*  CM: 3.0 (1.5-4.3)*  SMA; 1.3 (0.9-2.0)* | Not determined | UM < CM, SMA  S < NS:  CM S < CM NS  SMA S < SMA NS |  |
| Conroy et al. (2012) | Malawi, N=155  Retrospective case-control study  CM-N: 3.6 (2.6-6.6)*  CM-R: 2.7 (2.1-3.7)* | CM-N > CM-R | CM-N < CM-R  CM-R S < CM-R NS |  |
| Moxon et al. (2014) | Malawi, N=226  Prospective case-control study  HC: 4.6 (3.7–5.7)**  UM: 4.7 (4.1–5.3)**  CM: 4.1(3.3–5.2)** | Not determined | HC (0.23) < UM (0.58), CM (1.54)*** (enrolment);  Still increased after 4 weeks in UM (0.32) compared to HC (0.23)  50% patients lost-to-follow-up |  |
| Weinberg et al. (2014) | Tanzania, N=211  Prospective cohort study  HC: 8 (7 –8)*  MSM: 5 (4–6)*  SM: 5 (4–8)*  CM: 4 (4–5)* | Not determined | HC (1.1) < MSM (2.0) and SM (2.3) < CM (3.7) |  |
| Abdi et al. (2014) | Kenya, N=213  Retrospective cohort study  UM: 2.7 (1.8-3.3)*  SM: 3.9 (3-6.2)* | Not determined | UM < SM |  |
| Rubach et al. (2015) | Tanzania, N=259  Prospective observational study  HC:12.8 (2.6–3.2)**  UM: 3.7 (3.3-2.1)**  CM: 4.3 (4.6)**  NMC: 2.4 (1.9-2.8)** | Not determined | HC (0.815), UM (1.88), NMC (1.15) < CM (3.45)* |  |

**CM,** cerebral malaria (WH’s definition); **CM-N,** cerebral malaria without retinopathy; **CM-R**, cerebral malaria with retinopathy; **HC**, healthy control; **MSM,** moderate severe malaria: fever within the preceding 48 h, with >1,000 asexual *P. falciparum* parasites/L, with no WHO warning signs or criteria for SM and a requirement for inpatient parenteral therapy because of an inability to tolerate oral treatment; **NMC**, non-malaria central nervous system conditions*;* **NS**, non survivors; **S**, survivors; **SM,** severe malaria; **SMA**, severe malarial anaemia; **UM,** uncomplicated malaria.

*Median, (IQR)

** Mean (95% CI)

*** Geometric mean
