# Supplementary material for: Systematic review of the role of angiopoietin-1 and angiopoietin-2 in Plasmodium species infections: biomarkers or therapeutic targets?
Source: Malar J. 2016 Dec 1;15:581. doi: 10.1186/s12936-016-1624-8 (PMC5134107; doi:10.1186/s12936-016-1624-8)
Supplement: Supplementary file 6 — Additional file 6. Studies on the effect of anti-inflammatory interventions on Ang-1 and Ang-2 levels during Plasmodium spp. infections in humans and mice. [file 12936_2016_1624_MOESM6_ESM.docx]

**Additional file 6 – Studies on the effect of anti-inflammatory interventions on Ang-1 and Ang-2 levels during *Plasmodium spp* infections in humans and mice.**

|  | **References** | **Population, N** | **Intervention** | **Ang-1 or Ang-2** | **Ang2/Ang1** | **Survival rate** |
| --- | --- | --- | --- | --- | --- | --- |
| **Mice, *PbA* infected** | Finney et al. (2011) | C57BL/6, 6-8 weeks, female | FTY720 or LX2931administration i.p. 1 day prior to infection or 1, 3 or 5 days after infection. All mice received artesunate once 5 days after infection. | FTY720: Ang-1, higher in prophylactic treated mice vs untreated mice. No effect in mice treated after infection. |  | FTY720: increased in mice treated prophylactic or treated 1 day after infection. Not increased when treatment was given 3 or 5 days post-infection.  LX2931: no increase in survival. |
|  | Serghides et al. (2011) | C57BL/6, 7-9 weeks, male | Inhalation of NO or placebo (air) 1 day prior to infection or 3 or 5.5 days post infection  Nitrite in drinking water day of infection compared with drinking water without additions.  All mice treated 3 or 5 days after infection received also artesunate. | Ang-1: higher in mice receiving prophylactic iNO compared to air treated mice. | Lower in mice receiving prophylactic iNO compared to air treated mice. | iNO: increased in both prior to and post infection treated mice compared to control mice.    Nitrite: increased in nitrite treated mice compared to control mice. |
|  | Kim et al. (2014) | C57BL/6, C5aR-/-, C5L2-/-, 7-11 weeks, male, female | Infection of all groups of mice. | Ang-1 protein level: Higher in C5aR-/- compared to WT; No difference between C5L2-/- and WT  Ang-2 mRNA: Lower in C5aR-/- compared to WT; No difference between C5L2-/- and WT |  | Increased in C5aR-/- mice compared to WT.  No increase in C5L2-/- mice |
|  | Serghides et al. (2014) | C57BL/6, 7-8 weeks, female | Treatment with artesunate and adjuvant rosiglitazone or placebo 3 days post-infection or on onset CM signs (5-6 post infection). | Ang-1: uninfected mice = rosiglitazone+artesunate > artesunate (in blood) | Rosiglitazone < artesunate (in brain homogenates) | Increased in mice receiving rosiglitazone+artesunate compared to artesunate alone |
|  |  | BALB/C Ang-1del , BALB/C WT (Ang-1 sufficient) | Treatment with artesunate and rosiglitazone or artesunate alone, start day 5.5 post infection. | Ang-1: Ang-1^del^ mice produce 30-50% of the Ang-1 levels of Ang-1 sufficient mice. |  | Increased in rosiglitazone treated Ang-1 sufficient mice but not in Ang-1del mice. |
| **Humans, *Pf.* infected** | Serghides et al. (2014) | Thailand, N=140  Randomized double-blind  placebo controlled trial  Age in years (mean, (SD))  Rosiglitazone: 26,5 (10.3)  Placebo: 26.1 (9,5) | Atovaquone-proguanil+rosiglitazone vs atovaquone-proguanil+placebo |  | Decreased 3 days after start treatment for rosiglitazone treated humans vs placebo treated humans. |  |
|  | Mwanga-Amumpair et al (2015) | Uganda, N=92  Randomized open label clinical trial, Phase II  Age in years (mean (SD)):  N_2:_ 2.8 (1.9)  NO:3.5 (1.9) | Adjuvant iNO or iN_2_ treatment (for 48-120h) | Ang-1: Increased in NO group over 48h, not in N_2_ group. No intergroup differences  Ang-2: Decreased in NO and N_2_ group over 48h. No intergroup differences. | Decreased in NO and N_2_ group over 48h. No intergroup differences | No intergroup differences. |
|  | Hawkes et al (2015) | Uganda, N=180  Randomized placebo-controlled, blinded trial  Age in years (median (IQR)):  iNO:2.0 (1.0–3.0);  Placebo: 2.0 (1.0–3.0) | Adjuvant iNO or air placebo treatment. (72h) | Ang-2: no significant differences between groups during 72h of hospitalization.  Ang-2 at admission S < NS |  |  |

**Ang-1**, angiopoietin-1; **Ang-2**, angiopoietin-2; **Ang-1^del^ mice**, mice with one Ang-1 allele; **CM**, cerebral malaria; **FTY720**: antagonist of Sphingosine-1-phosphate receptor; **iN2**, inhaled nitrogen; **iNO**, inhaled nitric oxide; **i.p**., intraperitoneal; **NS**, non survivors; ***PbA***, *Plasmodium Berghei* ANKA; ***Pf***. *Plasmodium falciparum*; r**osiglitazone**, PPARγ agonist, antidiabetic drugs in thiazolidinedione class, anti-inflammatory features; **S**, survivors; **WT**, wildtype; **-/-**, knock-out.
