# Supplementary material for: Systematic review of the role of angiopoietin-1 and angiopoietin-2 in Plasmodium species infections: biomarkers or therapeutic targets?
Source: Malar J. 2016 Dec 1;15:581. doi: 10.1186/s12936-016-1624-8 (PMC5134107; doi:10.1186/s12936-016-1624-8)
Supplement: Supplementary file 7 — Additional file 7. Ang-1 and Ang-2 as biomarkers for disease severity during human Plasmodium spp. infection. [file 12936_2016_1624_MOESM7_ESM.docx]

**Additional file 7** – **Ang-1 and Ang-2 as biomarkers for disease severity during human *Plasmodium spp.* infection.**

| **Study**  *species* | **Distinguishing** | **AUC Ang-1 (95% CI; p-value)**  **Sensitivity (95% CI); specificity (95% CI)** | **AUC Ang-2 (95% CI; p-value)**  **Sensitivity (95% CI); specificity (95% CI)** | **AUC Ang-2/Ang-1 (95% CI; p-value)**  **Sensitivity (95% CI); specificity (95% CI)** |
| --- | --- | --- | --- | --- |
| **Yeo et al. (2008, 2010 ( ref. 35))**  *P. falciparum* | SM S - SM NS |  | 0.84 (0.71-0.96)  Higher AUROC than lactate 0.63 (0.41-0.83), comparable AUROC with HRP2 0.86 (0.73-0.94) |  |
| **Lovegrove et al. (2009)**  **Thailand – adults**  *P. falciparum* | UM - CM | 1 (1-1; p<0.001)  100 (87-100); 100 (87-100) | 0.84 (0.72-0.95; p<0.001)  72 (52-86); 84 (65-94) | 1 (1-1; p<0.001)  100 (87-100); 100 (87-100) |
| **Uganda – children**  *P. falciparum* | UM - CM | 0.79 (0.71-0.86; p<0.001)  70 (58-79); 75(63-83) | 0.69 (0.60-0.78; p<0.001)  83 (72-90); 60 (48-71) | 0.78 (0.70-0.86; p<0.001)  73 (61-82); 70 (58-79) |
| **Conroy et al. (2009)**  *P. falciparum* | UM – SM  UM – CM  SM – CM  UM – SM and CM | 0.88 (p<0.001)  86 (71-94); 85 (76-92)  0.78 (p<0.001)  71 (61-80); 74(63-83)  0.74 (p<0.001)  72 (56-84); 66 (55-75)  -  71 (62-78); 77 (66-85) | 0.76; (p<0.001)  69 (53-82); 79 (68-87)  0.77 (p<0.001)  76 (66-84); 77 (66-85)  0.53 (p=0.663)  47 (32-63); 64 (54-74)  -  74 (66-81); 77 (66-85) | 0.86 (p<0.001)  81 (65-90); 83 (72-90)  0.82 (p<0.001)  78 (68-86); 83(72-90)  0.60 (p=0.084)  44 (30-60); 83 (74-89)  -  79 (71-85); 83 (74-89) |
| **Conroy et al. (2010)**  *P. falciparum* | CM-N - CM-R  UM - CM-R  CNS - CM-R | 0.64 (0.51-0.78; p=0.046)  68 (53-81); 44 (28-62)  0.96 (0.93-1; p≤0.05)  94 (80-98); 87 (73-94)  0.93 (0.88-0.99; p≤0.05)  88 (69-96); 87 (73-94) | 0.77 (0.65-0.89; p≤0.05)  71 (55-83); 72 (54-85)  0.65 (0.51-0.79; p=0.036)  63 (45-77); 71 (55-83)  0.71 (0.55-0.84; p≤0.05)  67 (47-82); 71 (55-83) | 0.74 (0.60-0.87; p=<0.05)  74 (58-85); 66 (47-80)  0.96 (0.93-1.0; p=<0.05)  94 (80-98); 95 (83-99)  0.93 (0.87-0.99; p=<0.05)  71 (51-85); 95 (83-99) |
| **Erdman et al (2011)**  *P. falciparum* | S – NS |  | 0.83 (0.75-0.90; p<0.01)  78 (56-93); 79 (68-87) |  |
| **Jain et al. (2011)**  *P. falciparum* | MM – CM NS  CM S – CM NS | 0.35  0.50 | 0.95  90; 90  0.76  83;64 | 0.90  86; 83  0.69  66;60 |
| **Conroy et al. (2012)**  *P. falciparum* | CM S - CM NS | 0.56 (0.47-0.65; p=0.20) | 0.71 (0.63-0.78; p≤0.0001)  AUROC not different from lactate 0.67 (0.59-0.78; p=0.002) |  |
| **Prapansilp et al. (2013)**  *P. falciparum* | S – NS |  | 0.73 (0.60-0.85)  AUROC not different from lactate 0.67 (0.53-0.81) | 0,66 (0.52-0.80)  AUROC not different from lactate (0.67 (0.53-0.81) |
| **Gomes et al. (2015)**  *P. vivax* | All patients  Platelet count <75000 µL  Platelet count >75000 µL |  | 0.67  78; 50  0.83  88; 67  0.53  80; 29 | 0.74  61; 84  0.88  75; 91  0.65  60; 68 |

**CM**, cerebral malaria; **CM-N**, cerebral malaria without retinopathy; **CM-R**, cerebral malaria with retinopathy; **CNS**, fever and decreased consciousness, not malaria **MM**, mild malaria; defined as parasitemia of < 25,000 parasites/μl and no evidence of severe malaria and no past history of mental/metabolic illness, tuberculosis, meningitis, or accidental head injury; **NS**, non-survivors; **S**, survivors; **SM**, severe malaria; **UM**, uncomplicated malaria.
